# Modified full-face snorkel mask as COVID-19 personal protective equipment: Quantitative results

**DOI:** 10.1016/j.ohx.2021.e00185

**Published:** 2021-02-25

**Authors:** Kyle Nicholson, Ashley Henke-Adams, Daniel M. Henke, Alexxai V. Kravitz, Hiram A. Gay

**Affiliations:** a McKelvey School of Engineering, Washington University, St. Louis, MO, United States; b Barnes Jewish Hospital, St. Louis, MO, United States; c Henke Engineering, LLC, Festus, MO, United States; d Departments of Psychiatry, Anesthesiology, and Neuroscience, Washington University School of Medicine, St. Louis, MO, United States; e Department of Radiation Oncology, Washington University School of Medicine, St. Louis, MO, United States

**Keywords:** SARS-CoV-2, 3D printed, Adapter, N95, P95, P100, Face shield, Quantitative fit testing, Fit factor, Commercial air filter, OSHA, 3M filter, Bayonet connection

## Abstract

The COVID-19 crisis has resulted in a shortage of personal protective equipment (PPE). Adapting commercially available full-faced snorkel masks has been proposed as an alternative to narrow the gap in PPE. An advantage of the full-faced snorkel mask design is that it serves two critical purposes: eye and face protection, and high quality air filtration to protect against SARS-CoV-2. We performed quantitative testing on various full-faced snorkel masks with 3D printed adapters that accept commercially available particulate filters, and report on a design that passed Occupational Safety and Health Administration (OSHA) full-facepiece respirator standards.

## Graphical abstract

**Figure:**
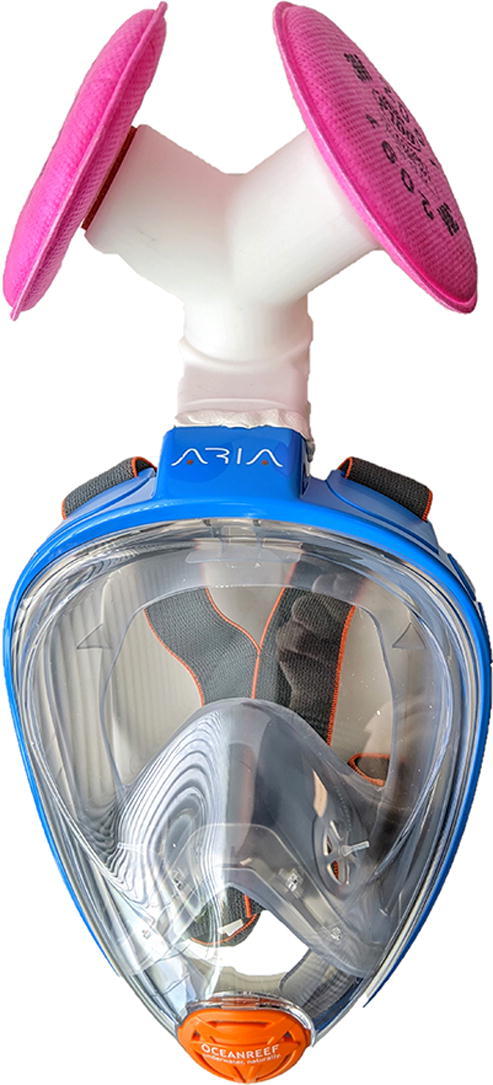

Specifications table

**Table:** 

Hardware name	Modified Ocean Reef Aria full-face snorkel mask as COVID-19 personal protective equipment (PPE)
Subject area	Medical
Hardware type	Personal Protective Equipment (PPE)
Open Source License	Creative Commons Attribution-ShareAlike license
Cost of Hardware	• Full face snorkel mask: recommend the Aria Classic ($69.95) or Aria QR+ ($89.95), the least expensive available. Unless the only option, avoid the Aria UNO as the strap design is more prone to user error and compromising seal • Sticky putty: $5.59 • Pair of 3M filters (< $15 for a pair). Unless the only option, avoid the clamshell design which will increase the costs and is more prone to user error • Pair of 3M 3PRG7 inhalation port gaskets: $4.02 • 3D printed adapter: variable cost
Source File Repository	DOI: https://doi.org/10.17605/OSF.IO/YXBTR

## 1 Hardware in context

The COVID-19 crisis has resulted in a shortage of personal protective equipment (PPE) [1]. Adapting commercially available full-faced snorkel masks has been proposed as an alternative to narrow the gap in PPE [2]. An advantage of the full-faced snorkel mask design is that it serves two critical purposes: eye and face protection, and high quality air filtration to protect against SARS-CoV-2.

Multiple snorkel mask solutions have been circulated online, but none to our knowledge have undergone and passed rigorous quantitative testing [3], [4], [5]. One of the snorkel mask modifications explored using a ventilator filter, and although promising failed the quantitative testing [5]. Due to the smaller size of ventilator filters, it is unknown the long term breathability of these filters without the assistance of a ventilator. Others have proposed custom-made 3D-printed face masks to substitute N95 masks without protection of the entire face [6], [7]. We performed quantitative testing on various full-faced snorkel masks with 3D printed adapters that accept commercially available particulate filters, and report on a design that passed Occupational Safety and Health Administration (OSHA) full-facepiece respirator standards.

## 2 Hardware description

**Fig. 1:**
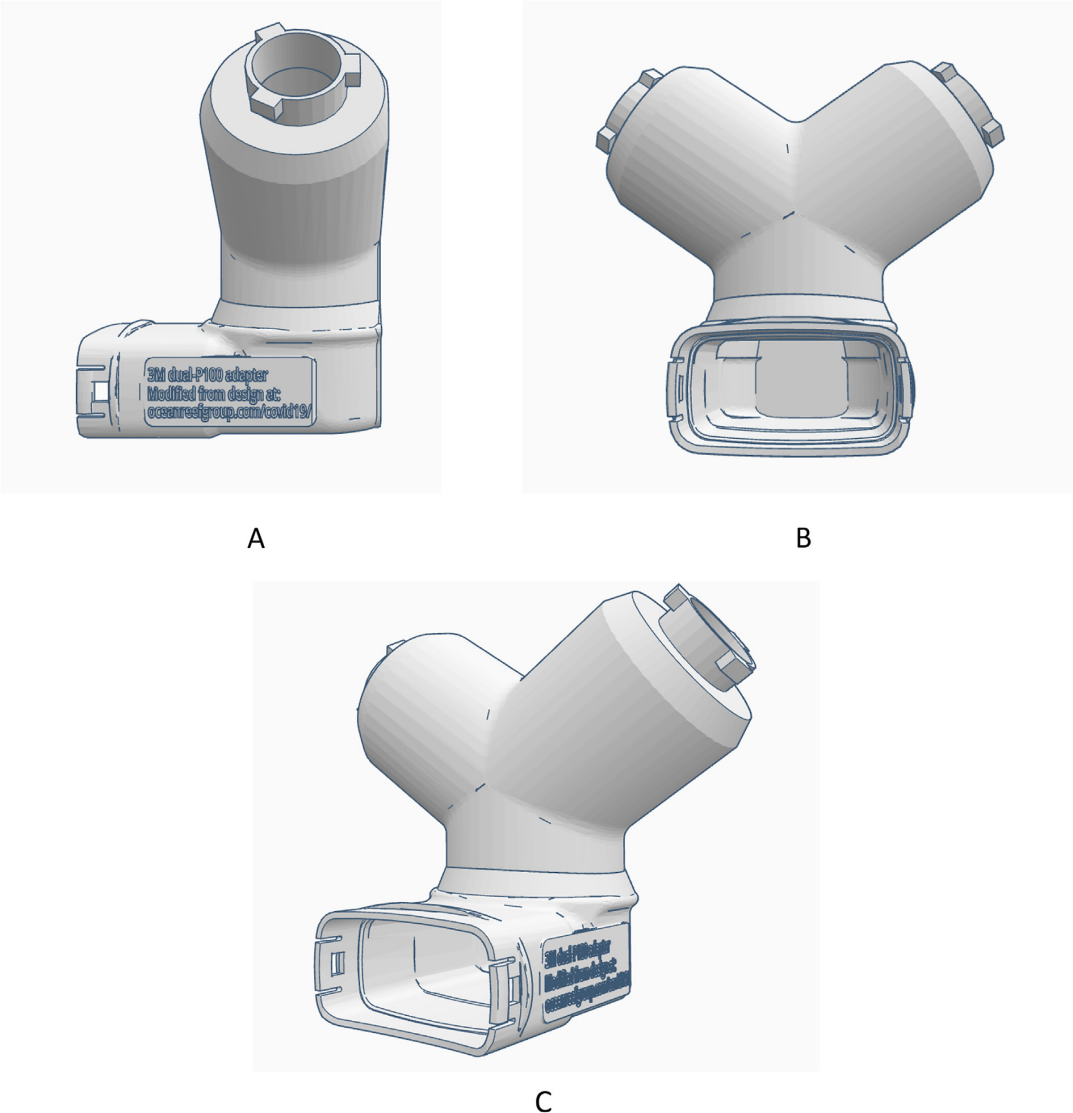
CAD drawing **A)** Lateral view **B)** Front view **C)** Oblique view.
• Any 3M filter with a bayonet connection, some of which we tested (Fig. 2), could be used and provide multiple filtration options depending on filter availability and cost.

• An advantage of the full-faced snorkel mask design is that it serves two critical purposes: eye and face protection, and high quality air filtration to protect against SARS-CoV-2.
• The Aria full face snorkel masks minimize fogging problems and CO_2_ rebreathing, are readily available and less costly than commercial 3M full face respirators, and the manufacturer has a clear sizing guide based on the distance from the nose bridge to the bottom of the chin to ensure an optimal fit.
• 3D printing is a cost-effective readily available technology that can be used to print the adapters (Fig. 1).

**Fig. 2:**
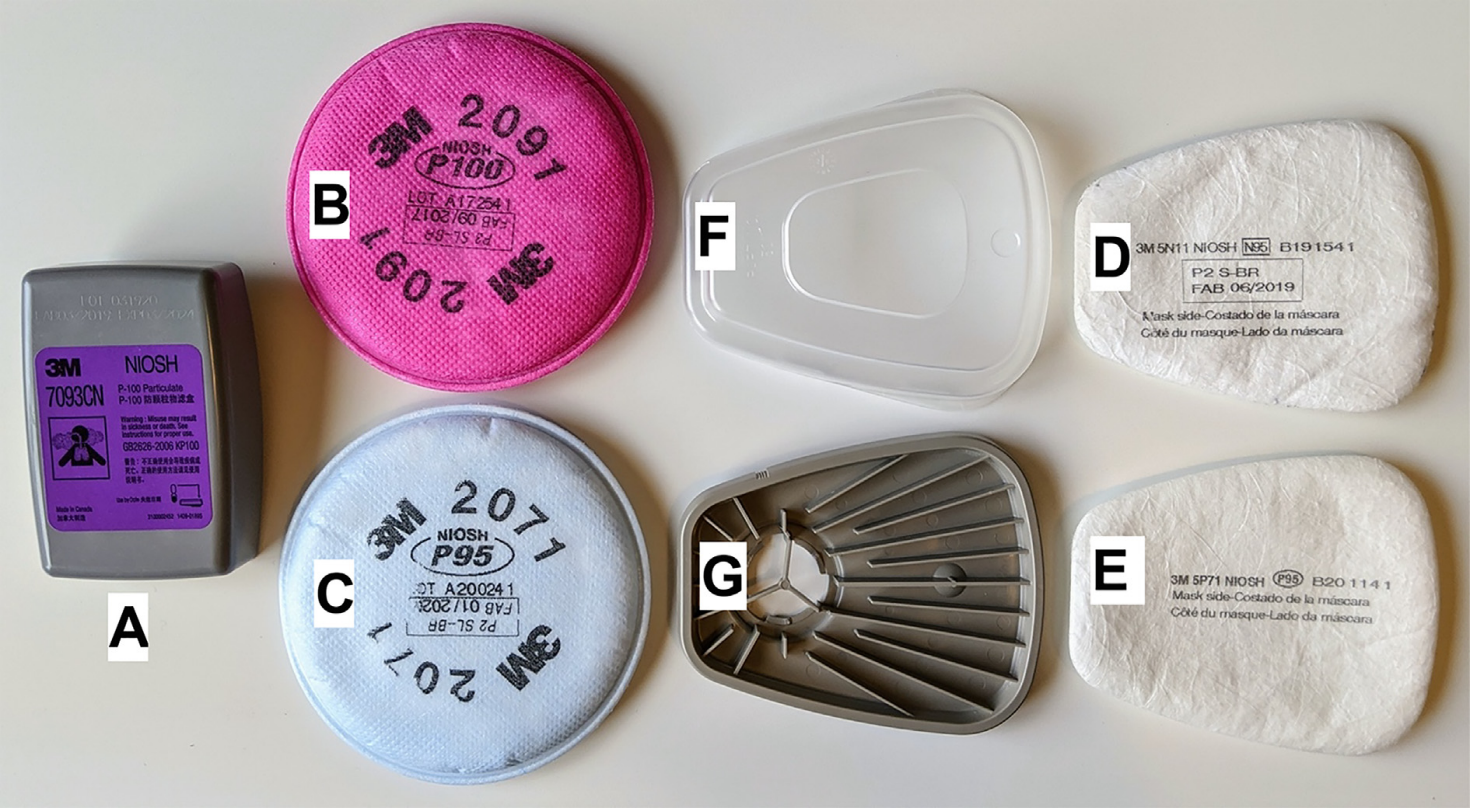
3M filters: **A)** 7093CN P100 **B)** 2091 P100 **C)** 2071 P95 **D)** 5N11 N95 **E)** 5P71 P95 **F)** 501 filter retainer **G)** 603 filter adapter.

### 2.1 Modified full-face snorkel mask hardware components

#### 2.1.1 Full face snorkel-mask

Aria full face snorkel masks (Ocean Reef, Inc., California, USA) were tested. We tested three Aria QR+ (Medium/Large), one Aria Classic (Small/Medium), one Aria Classic (Large/Extra Large), and one Aria UNO (S/M) full-faced snorkel masks*.*

#### 2.1.2 3D printed adapter

The adapter was a modification of the APA – Aria Protection Adapter available at the Ocean Reef website (https://oceanreefgroup.com/covid19/). The original adapter was designed to accept a 40 mm particulate air filter with 1/7 in. thread. The design was modified to accept two bayonet connection 3M filters. We made an earlier prototype with a single filter but found it required too much breathing effort to use for long durations. Fig. 1 shows a CAD drawing of the adapter. The design reduced the printing material required compared to a vertical alignment of the filter.

#### 2.1.3 Filters

We tested the following filters:

A. 3M 7093CN particulate filter with 99.97% filter efficiency meeting NIOSH P100-series test criteria
B. 3M 2091 P100 particulate filter with 99.97% filter efficiency meeting NIOSH P100-series test criteria
C. 3M 2071 P95 particulate filter with 95% filter efficiency meeting NIOSH P95-series test criteria
D. 3M 5N11 particulate filter with 95% filter efficiency meeting NIOSH N95-series test criteria encased in the 3M filter adapter 603 and filter retainer 501
E. 3M 5P71 particulate filter with 95% filter efficiency meeting NIOSH P95-series test criteria encased in the 3M filter adapter 603 and filter retainer 501

Under 42 CFR 84 (this USA Code of Federal Regulations (CFR) rule addresses NIOSH and the Department of Labor/Mine Safety and Health Administration certification requirements for respiratory protective devices) these filters are challenged with laboratory generated aerosols, which are the most difficult size to capture: particles with approximately 0.3 μm mass median aerodynamic diameter (MMAD). Particles both smaller and larger than this size are captured at a higher efficiency, and most aerosols found in the workplace are larger than 0.3 μm MMAD. By testing with the most penetrating particle size it can be reliably predicted that filters will perform at their certified efficiency level (95%, 99%, or 99.97%) or better when used against aerosols present in the workplace. [8]

Under 42 CFR 84 the test aerosol used depends on the filter classification: the N-series filters are tested with solid sodium chloride (NaCl) particles, and R- and P-series filters with dioctyl phthalate (DOP), an oil. Therefore, N-series filters are approved for protection against non-oil aerosols only, and R- and P-series filters are approved for both oil and non-oil aerosols. [8]

Most viruses have a diameter between 0.02 μm and 0.3 μm. [9] The SARS-CoV-2 virus has a diameter between 0.06 μm to 140 μm. [10] Despite the small size of viruses, viral droplets typically may range from 4.44 μm (2 standard deviations below mean size) with sneezing to 23.5 μm (2 standard deviations above mean size) with breathing. [11] In summary, these filters are rated based on the worst-case scenario particles with 0.3 μm MMAD and the aerosol particles exhaled by a person are typically an order of magnitude larger.

### 2.2 Drilled full face snorkel-mask grommet fit testing method hardware components

This method requires perforating the snorkel mask and attaching a PortaCount grommet for testing N95 masks to match the diameter of the PortaCount air sampling tubing. This method is detailed in Section 5.2. The advantage of this methodology is that it is very convenient for quickly prototyping adapters, but the disadvantage is that it permanently damages the mask.

### 2.3 Universal intact full face snorkel-mask fit testing method hardware components

This requires modifying a 3D adapter and inserting 3 mm (1/8″) inner diameter, and 5 mm (3/16″) outer diameter PVC clear vinyl tubing (part number A19051400ux0443, Uxcell, Hong Kong, China), and using a 1/8″ barbed nylon straight coupler (model C0-2BN, Eldon James, Denver, CO) or similar connector to create an airtight connection with the PortaCount clear tubing. This method is detailed in Section 5.3. The advantage of this method is that it does not damage the mask, and is ideal to test users once the adapter design has been finalized.

## 3 Design files

### 3.1 Design files summary

**Table:** 

Design file name	File type	Open source license	Location of the file
OceanReef filter adapter.stl	.stl	Creative Commons Attribution-ShareAlike license	https://www.tinkercad.com/things/7dZnjwUKZRr (Supplementary .stl file)

This is a 3D file designed in the free program TinkerCAD. It facilitates the use of commercially available 3M respirator filters with a commercial full-face snorkel mask. We release both the 3D printing .stl file and the design files as open-source so users are able to modify this design to fit filters from different manufacturers, depending on the current supply.

## 4 Bill of materials

**Table:** 

Component	Number	Cost per unit – USD	Total cost - USD	Manufacturer/ Source of materials	Material type
**Full face snorkel masks**					
Aria QR+	1	$89.95	$89.95	Ocean Reef, Inc./Amazon	NA
Aria Classic	1	$69.95	$69.95	Ocean Reef, Inc./Amazon	NA
Aria UNO*	1	$39.95–$49.95	$39.95–$49.95	Ocean Reef, Inc./Amazon	NA
Optical Lens Support	1	$32.95	$32.95	Ocean Reef, Inc./Amazon	
**Full face respirator**					
3M 6800		$165.85–$214.95	$165.85–$214.95	3M/Jon-Don, Global Industrial, Uline	NA
**3M filters**					
7093CN P100	1 pair	$9.07–$13.16	$9.07–$13.16	3M/Jon-Don, Global Industrial, Uline	NA
2091 P100	1 pair	$8.30 – $10.75	$8.30 – $10.75	3M/Jon-Don, Global Industrial, Uline	NA
2071 P95	1 pair	$6.60–$7	$6.60–$7	3M/Global Industrial, Uline	NA
501 filter retainer	1 pair	$4–$5.10	$4 - $5.10	3M/Jon-Don, Uline, Amazon	NA
603 filter adapter	1 pair	$12.99	$12.99	3M/Amazon	NA
5N11 N95	1 pair	$4–$4.59	$4–$4.59	3M/Global Industrial, Uline	NA
5P71 P95	1 pair	$4.22–$5.59	$4.22–$5.59	3 M/Jon-Don, Global Industrial, Uline	NA
**3D printed adapter**					
black polylactic acid (PLA + )	1	∼$3	∼$3	eSUN	black polylactic acid (PLA + )
XTC-3D	24 oz	$ 25.86–$ 28.98	$ 25.86 –$ 28.98	Smooth-On, Inc./Amazon	epoxy
white Somos GP resin	1	∼$12	∼$12	DSM	stereolithography resin
white E-Rigid PU	1	∼$10	∼$10	EnvisionTEC	polyurethane
3PRG7 gasket	1 pair	$4.02	$4.02	3M /Grainger	NA
Museum & Gallery Sticky putty	3 oz	$5.59	$5.59	Alcolin/Amazon	NA
**Fit testing**					
PortaCount 8030 Particle generator 8026		$160 1 day rent^**^	$160 1 day rent^**^	TSI/Raeco Rents	NA
SurfacePro or Windows 7, 8, or 10 computer		varies	varies (rent or buy)	Microsoft or other vendor	NA
Nellcor OxiMax N-65 Pulse Oximeter		Varies, discontinued	varies	Medtronic or other vendor	NA
*Drilled full face snorkel-mask grommet method*					
PortaCount grommet^***^	1	< $1	$205 (Kit SKU: 8025-N95)	TSI/TSI	NA
*Universal intact full face snorkel-mask method*					
PVC clear vinyl tubing, A19051400ux0443	< 60 cm	< $4	$13.59 (10 m)	Uxcell/Amazon	Vinyl
1/8″ barbed nylon straight coupler, model C0-2BN	1	$1.40	$1.40 (package of 10, $13.94)	Eldon James/Amazon	Nylon

*the strap design is more prone to user error and compromising seal

^**^Receive Friday and ship out Monday morning when UPS opens to maximize 1 day rent

^***^The fit test probe kit for disposable facepieces 8025-N95 has more than 100 grommets and a device for perforating N95 masks not necessary for this project. Recommend the universal intact full face snorkel-mask method as it is less expensive.

## 5 Build instructions

In this section we will describe the 3D printed adapter as well as two fit testing methods. The first method described in Section 5.2 is ideal for rapid prototyping of adapters, but requires drilling a hole permanently damages the mask. Once the final adapter design is determined, we recommend the second method described in Section 5.3 as it doesn’t damage the mask and can be used to test any number of individuals.

### 5.1 3D printed adapter

**Fig. 3:**
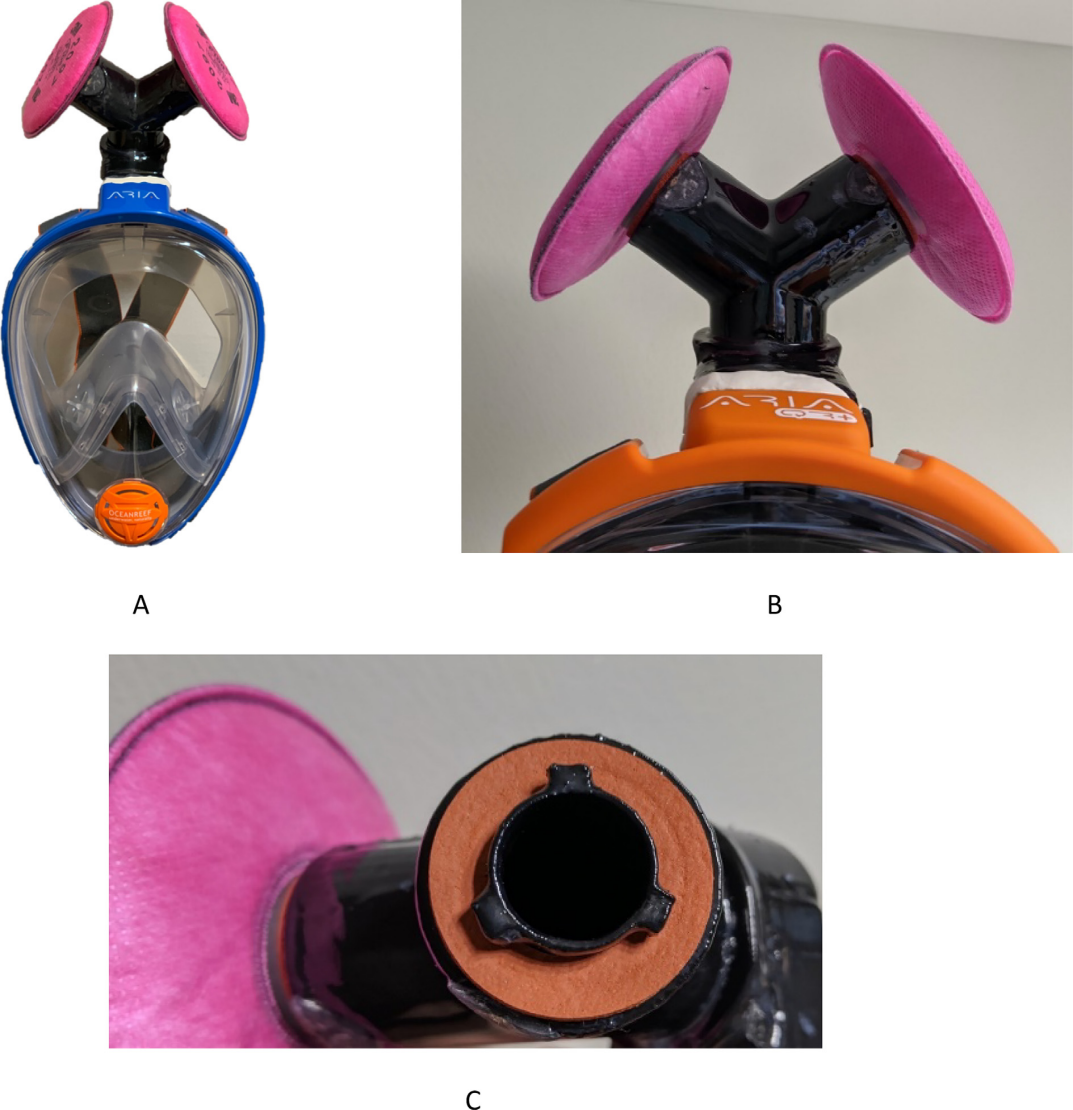
**A)** Snorkel mask with adapter and 3M 2091 P100 filters **B)** PLA adapter with filters attached **C)** View of the inhalation port with 3M gasket.

The first adapter prototype we tested was created with fused deposition modeling (FDM) black polylactic acid (PLA+, eSUN) print (Fig. 3). PLA prints are porous and without an airtight coating they will not work in an air filtration application. Two coats of XTC-3D (Smooth-On, Inc., Pennsylvania, USA) were applied to make it airtight. For detailed instructions on how to correctly prepare, apply, and cure the product see the manufacturer’s website: XTC-3D™ Product Information | Smooth-On, Inc. (smooth-on.com). One 3M 3PRG7 (3M, St. Paul, MN) inhalation port gasket was placed at each of the two inhalation ports.

Fused Deposition Modeling (FDM) is a common method for 3D printing, in which a continuous thermoplastic filament (in this case PLA) is heated to its melting point and used to build the part. In this process, the 3D design file is “sliced” by the computer into thin layers (0.2 mm), and the 3D printer deposits each layer onto the build platform by moving the print head in the X and Y direction while extruding the thermoplastic filament from the print nozzle. After each layer is deposited, the build platform is moved down by the thickness of one layer (0.2 mm) and the next layer is deposited. This process continues until the full part has been “grown” on the build platform. While other processes such as Selective Laser Sintering or StereoLithography printing can produce 3D prints with higher resolution, FDM is still commonly used because the machines and filament are much cheaper than those used in other processes.

There are several parameters that can be set for FDM printing, including layer height, infill density, and print speed. In general, thinner layers and slower print speeds will produce higher quality parts, although there are limits to the layer height and speeds that can be achieved with FDM printers. For the current parts, we printed with a 0.2 mm layer height, with a filament extrusion speed of 40 mm/sec, and with an infill density of 80%. These parameters were at the highest quality our printer can produce, and in our experience trying to push these parameters further can result in failed prints and other problems.

Sticky putty (Alcolin, Cape Town, South Africa) was used to ensure an airtight seal between the mask and the snorkel connector. Make sure the putty covers both the hard plastic snorkel connector circumferentially and the soft plastic covering it since air can leak through these two areas. Finally, one 2091 P100 filter was attached at each of the two inhalation ports although other bayonet connection 3M filters can be used, and we tested various others (Fig. 2).

We experimented with several prototypes in the development of this setup. Critical modifications were sealing the 3D printed PLA adapter to make it airtight, and sealing the connection between the 3D printed adapter and the mask with putty. We cannot overemphasize that with an airtight adapter the weakest link will be the connection between the adapter and the mask, and without proper sealing it will not work. It is possible that with a higher resolution printer the PLA print may not have needed to be sealed with the epoxy compound, but we were unable to test this. This sealing step also may not be necessary if different manufacturing processes or materials are used to create the adapter. To test this, we also printed the adapter in E-Rigid PU in white (EnvisionTEC), which provided an airtight seal. We also printed the adapter with stereo-lithography process in white Somos GP resin (DSM), which did not need any additional sealing. However, these prints were much more expensive to produce, and are not as accessible as FDM printing in PLA. The ideal printing process and material will depend on what is available to the end user, but care should be taken to ensure the material is airtight and doesn’t release unpleasant odors or potentially harmful volatile compounds to the user.

### 5.2 Drilled full face snorkel-mask with grommet fit testing setup

**Fig. 4:**
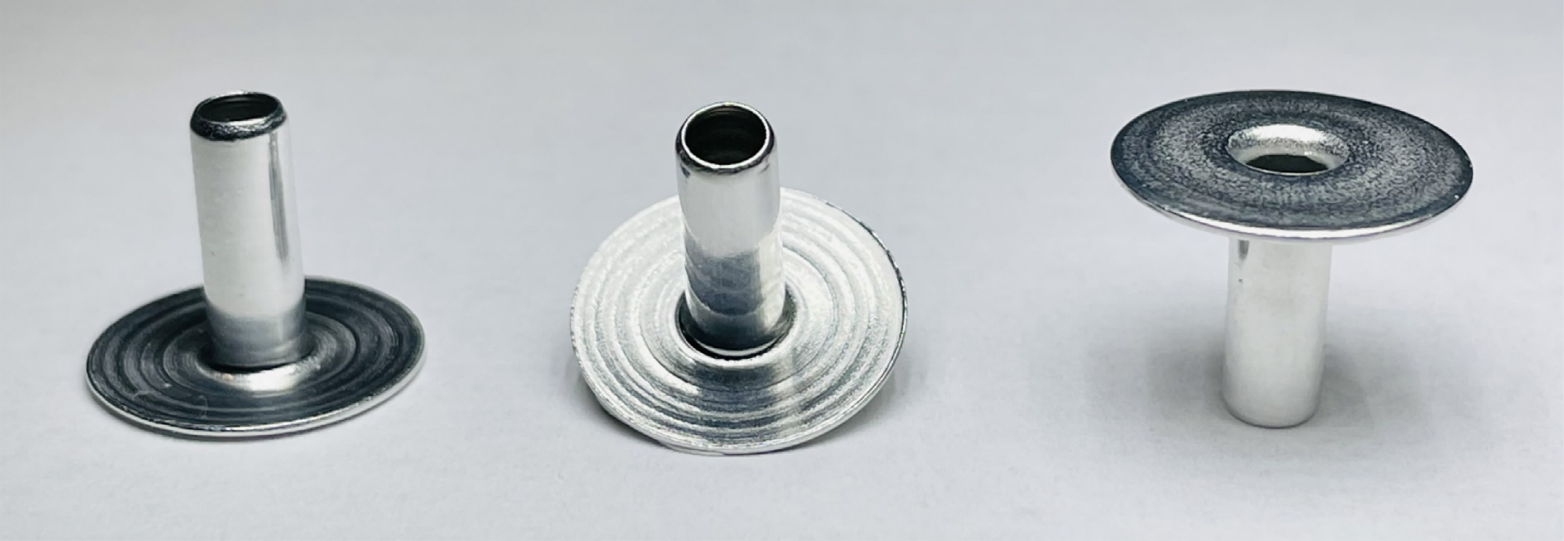
Three different views of the metal grommet. The flat disk is glued and sealed to the inside of the mask. Sealant should also be applied to the grommet’s cylindrical portion going through the drilled hole, and finally the exterior of the mask should be sealed. Select a large enough flat section inside the mask for an optimal seal as the mask has some curved surfaces.

**Fig. 5:**
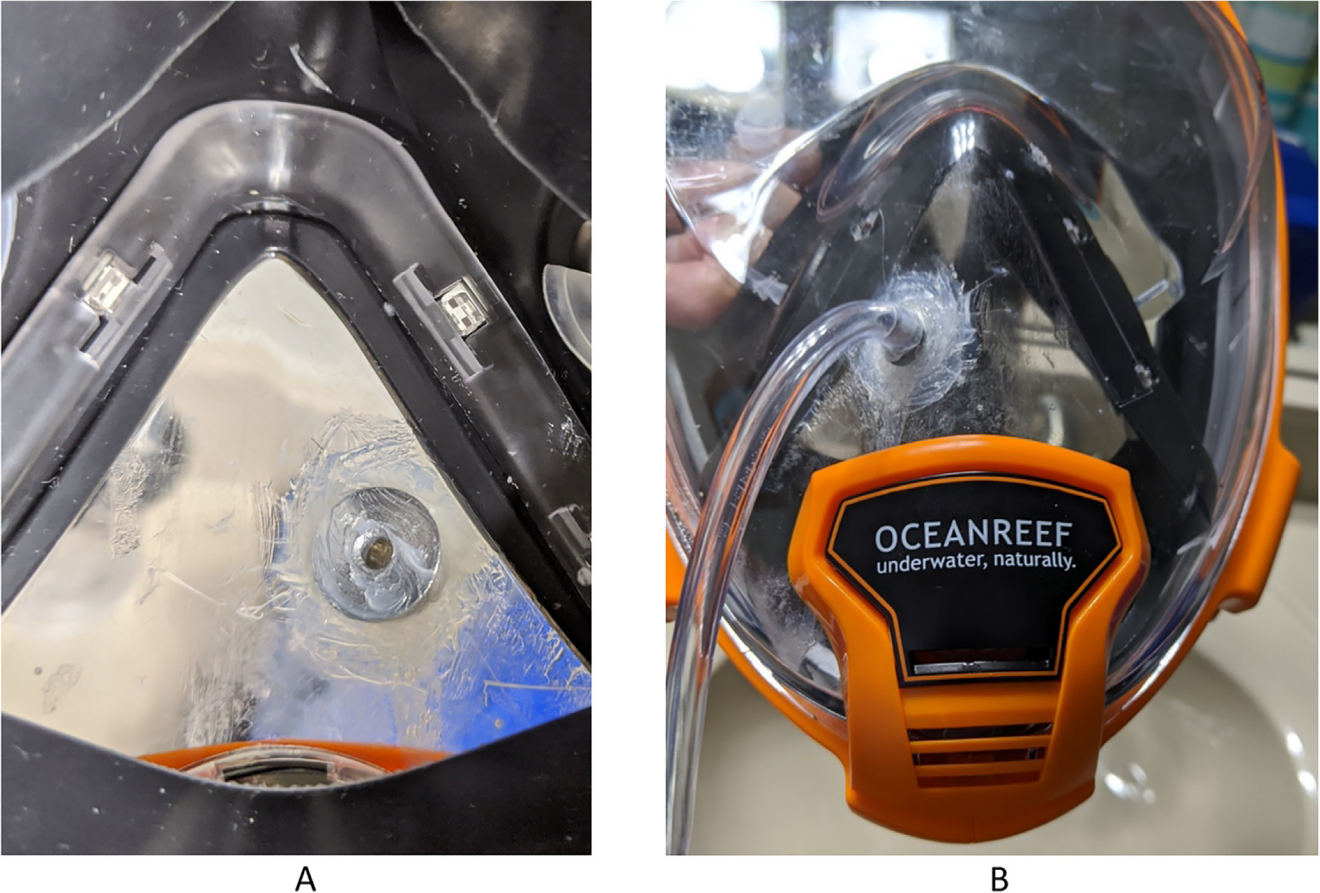
**A)** The metal grommet is glued and sealed inside of the mask. **B)** The outside and drilled surfaces of the mask are also sealed to eliminate potential air leaks. Extend the sealant beyond the grommet’s edge for a better seal. Even a pinhole leak could negatively impact the performance of the mask. Air sampling is inside the mouth/nose compartment. The PortaCount’s testing clear tube was secured and sealed with superglue. Wait until the fumes of the superglue dissipate to use the mask.

A hole was drilled into the mouth/nose mask compartment and a PortaCount grommet (Fig. 4) for testing N95 masks was inserted (Fig. 5). Silicone was added to seal the external and internal surfaces. Superglue was used to secure and seal the contact between the metal grommet and plastic tube to reduce air leaks that can impair accurate quantification of the fit factor. The PortaCount’s testing clear tube was secured and sealed with superglue. Allow enough time for the superglue and silicone to set and minimize inhaling any irritating fumes.

### 5.3 Modified adapter with intact full face snorkel-mask fit testing setup

1. This adapter, after proper disinfection, can be used to fit test multiple users.
2. Drill a 5 mm hole in the lower center back of the adapter (Fig. 6).

**Fig. 6:**
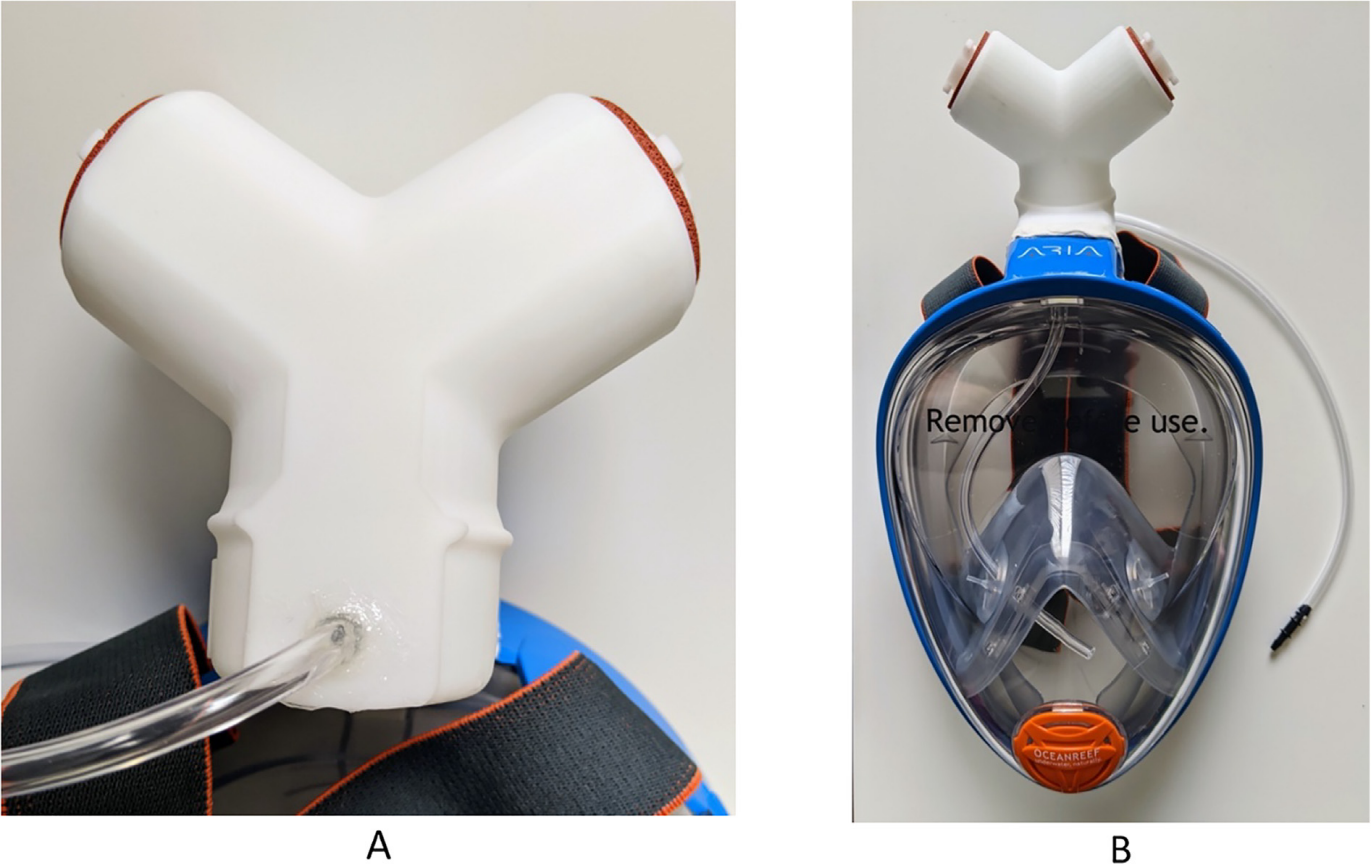
**A)** Fit test adapter in Somos GP resin (DSM) with PVC clear vinyl tubing going into the hole in the lower center back of adapter sealed with superglue **B)** PVC clear vinyl tubing going down the face compartment of the ARIA Classic snorkel mask, through mushroom valve and into the mouth/nose compartment. The black barbed nylon straight coupler connects to the PortaCount clear tube.
3. Insert the 3 mm (1/8″) inner diameter, and 5 mm (3/16″) outer diameter PVC clear vinyl tubing (part number A19051400ux0443, Uxcell, Hong Kong, China).
4. Feed the tubing through the hole in the adapter (seal with superglue to hold it firmly), into the middle air channel of the snorkel connector, down into the face compartment of the mask, through either the left or right mushroom valve, and into the mouth/nose compartment.
5. Use a 1/8″ barbed nylon straight coupler (model C0-2BN, Eldon James, Denver, CO) or similar connector to create an airtight connection with the PortaCount clear tubing.
6. When finishing the testing, verify the mushroom valve is back in its proper position which will help minimize CO_2_ rebreathing and facilitate CO_2_ exhaust.

## 6 Operation instructions

There are two recommended configurations of the snorkel mask depending on the situation:

1. **Snorkel mask duct tape configuration:** In this setup the front plastic cover is removed as shown in this video (skip to 1 min): https://www.youtube.com/watch?v=ewrsJ4lTgj4 (Supplementary Video 1)
  Supplementary video 1, Transforming Snorkeling Mask into non certified PPE - APA - FIRST CONCEPT! UNOFFICIAL!
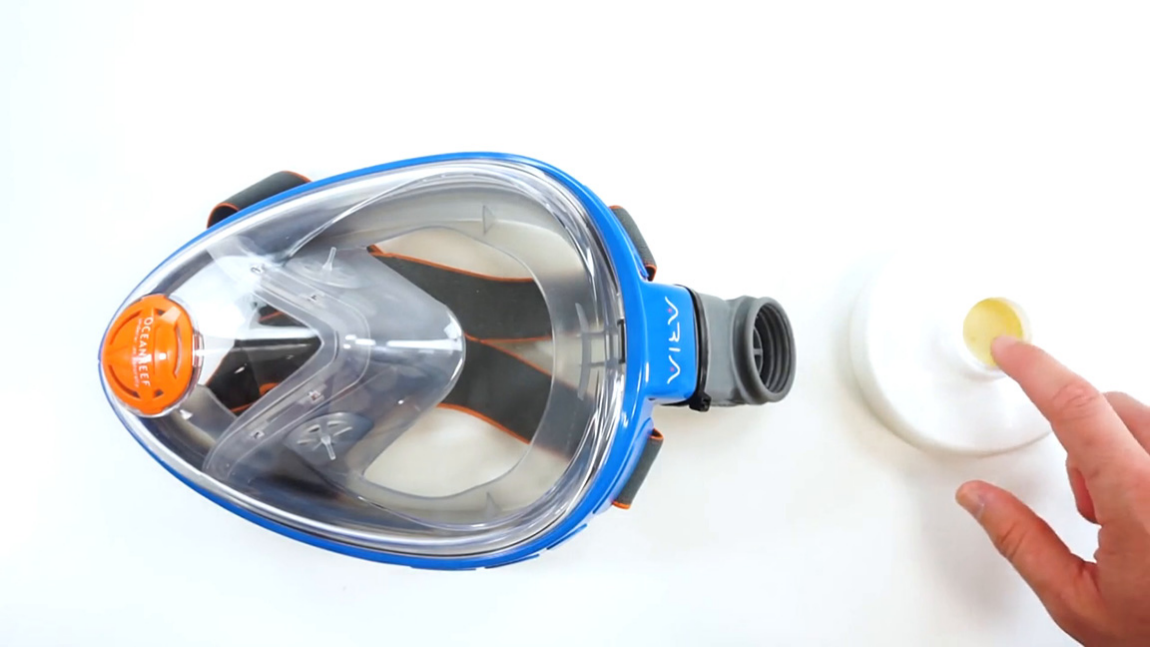
  The mushroom valve by the mouth is also removed. Two square pieces of duct tape cover the resulting opening. This configuration will protect both the user and others as the inhaled and exhaled air are filtered, but may be more uncomfortable for prolonged periods of time as the humidity inside the mask and the exhalation effort will be greater than the next configuration.
2. **Snorkel mask no modifications:** The snorkel mask is unaltered. **This configuration will only protect the user** as most of the exhaled air will exit unfiltered through the mushroom valve in the mouth’s exhaust port. This configuration may be more comfortable over extended periods of time as the humidity inside the mask and exhalation effort will be less than in the previous configuration. All things being equal, this configuration will achieve lower fit factors than the previous one, but when used properly should pass OSHA standards.
  We provide snorkel mask assembly video files for this snorkel mask configuration both in **standard definition** https://drive.google.com/file/d/17lWQb8a2bHQQulfc9uw0IteZ2j52ag1k/view?usp=sharing (Supplementary Video 2)
  Supplementary video 2, Snorkel mask no modifications assembly, standard definition video
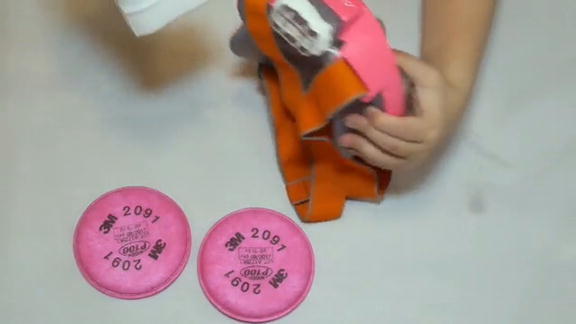
  and **high definition**
  https://drive.google.com/file/d/1CoCG7ouE2n8aq16O-wKkbQxgjyye3vkh/view?usp=sharing (Supplementary Video 3)
  Supplementary video 3, Snorkel mask no modifications assembly, high definition video
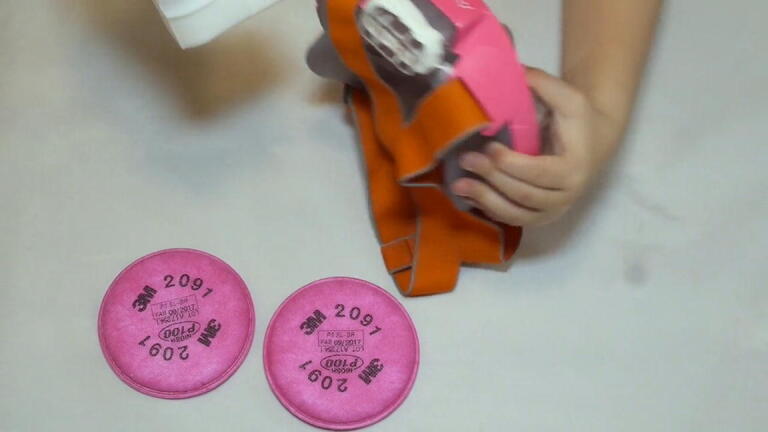
  illustrating the assembly of this configuration.

### 6.1 Initial full-faced snorkel mask setup

1. Write your name on the snorkel mask.
2. Perform hand hygiene using hand sanitizer.
3. Carefully inspect the disinfected snorkel mask, disinfected adapter and pair of filters (for dirt and physical damage to the filter or bayonet fitting). Verify the proper seating of the 3 mushroom valves inside the mask (2 in the mouth compartment and 1 in the exhalation port) and check for any damage. The 2 mouth compartment valves will help minimize CO_2_ rebreathing and facilitate CO_2_ exhaust.
4. Apply a continuous strip of putty bridging the hard plastic of the snorkel connector and the soft plastic seal wrapping around it from the mask (Fig. 7). Consider removing the black rubber seal (Fig. 7**a**) if you plan to attach and remove the filter multiple times as it can stretch and become loose. This is most relevant when planning to use it underwater in the future as a snorkel mask.

**Fig. 7:**
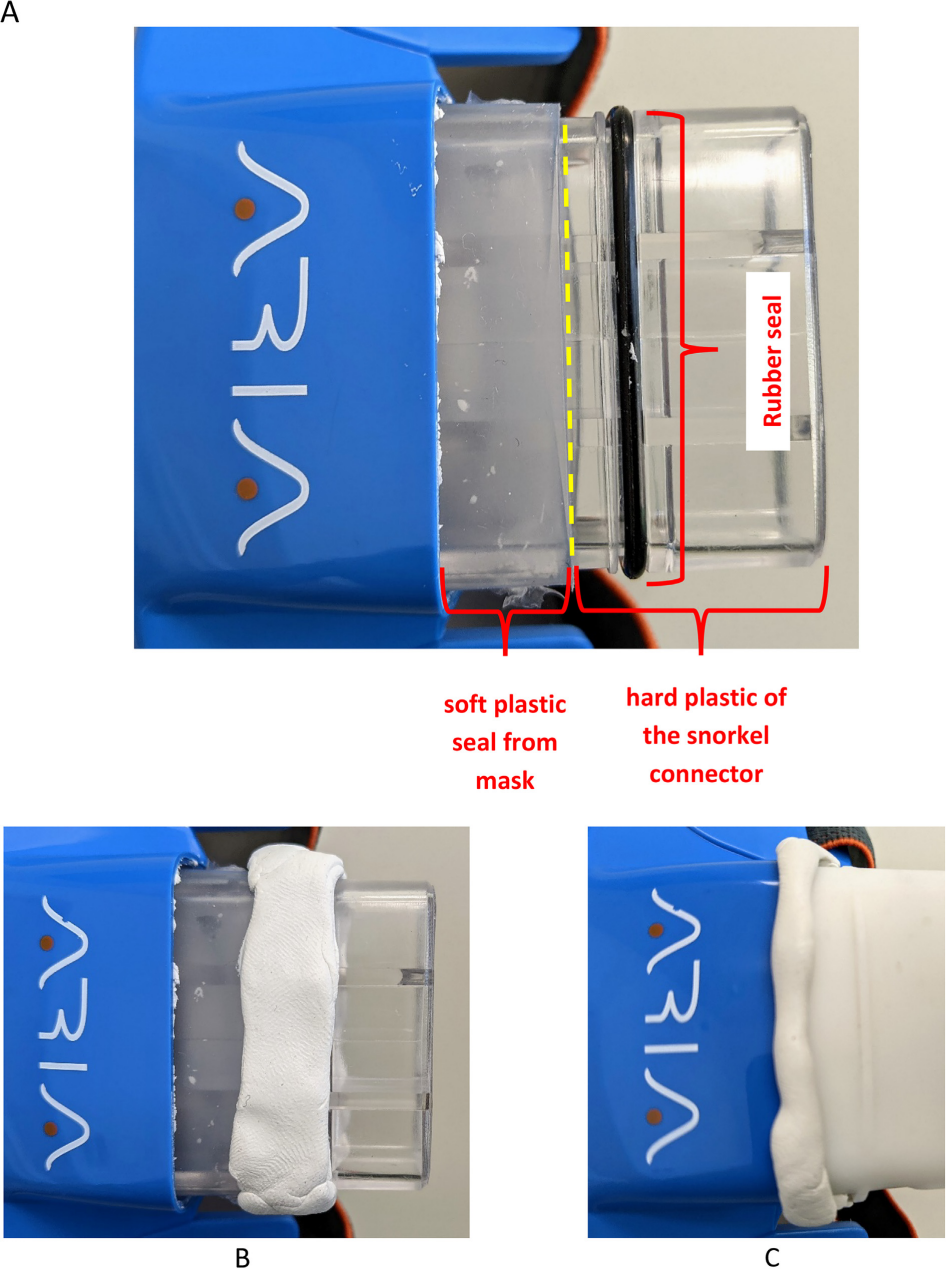
**A)** Photo of the soft plastic seal of the mask (extending to yellow intermittent line), black rubber seal, and hard plastic snorkel connector. **B)** Circumferential putty placement prior to inserting adapter. **C)** Adapter pushed in as far as possible and covering the soft plastic seal. Putty extruded circumferentially without any gaps to have an airtight and secure connection. (For interpretation of the references to colour in this figure legend, the reader is referred to the web version of this article.)
5. Push the 3D printed adapter gently and verify that some putty is extruded circumferentially without gaps. Push the adapter in as much as possible. It is important that the adapter covers the soft plastic seal and does not stop short of it as this could be a source of a large air leak and compromise the fit factor (Fig. 7).
6. Insert a 3M inhalation port gasket at each of the adapter’s bayonet connectors
7. Insert the pair of 3M filters per the manufacturer’s instructions. For most filters it involves aligning the filter bayonet fitting with the adapter’s bayonet fitting, and then rotating ¼ turn clockwise to attach.
8. Put on the mask fully. Adjust and tighten the straps as needed.
9. Perform a suction test: put the mask to your face, press it slightly while inhaling to create suction between the mask and your face. The mask should suction to your face. Try smiling and see if the movement of facial muscle breaks the seal. **IF MASK DOES NOT PASS THE SUCTION TEST, THE MASK SHOULD NOT BE USED.** Some factors that may affect the seal are: facial hair, eyeglasses, long hair, cosmetics, facial scars, face piercings, dentures, jewelry and facial structure. Any hair growth that contacts the sealing surfaces must be shaved clean. Long hair should we secured back to avoid interfering with the seal especially around the forehead. Eyeglasses will not permit a good seal and contact lenses should be used. Ocean Reef also provides an optical lens insert for the Aria full face snorkel masks (see **Bill of Materials,** Section 4).
10. Perform hand hygiene using hand sanitizer.

### 6.2 Full-faced snorkel mask fit testing

1. Follow the instructions in Section 6.1. Instead of the adapter, use the modified adapter for fit testing, and route the tubing as described in Section 5.3. This adapter should have been tested for air leaks previously.
2. Perform a qualitative or ideally quantitative fit testing per OSHA.
  o For quantitative testing, the adapter should have previously demonstrated a successful qualitative fit test with a fit factor of 500 or greater per OSHA for full-facepiece respirators. The fit factor is determined by taking the ratio of the average chamber concentration to the concentration measured inside the respirator for each test exercise except the grimace exercise. The calculation of the overall fit factor using individual exercise fit factors involves first converting the exercise fit factors to penetration values, determining the average, and then converting that result back to a fit factor. [12] This procedure is described in the following equation:
  o $\mathrm{OverallFitFactor}=\frac{N}{\frac{1}{FF1}+\frac{1}{FF2}+\frac{1}{FF3}+\frac{1}{FF4}+\frac{1}{FF5}+\frac{1}{FF6}+\frac{1}{FF7}}$
    Where:
    N = number of exercises = 7 (excluding grimace)
    Fit factors: FF1 = normal breathing; FF2 = deep breathing; FF3 = Head side to side; FF4 = Head up and down; FF5 = Talking; FF6 = Bending over; FF7 = Normal breathing
  o One key difference between a commercial mask with air filters connected to the mouth compartment is that the volume of air that needs to be purged is relatively small compared to the full face snorkel mask volume where the filters are attached superiorly to the face compartment. Therefore, it takes longer for the snorkel mask to reach particle equilibrium. When testing the snorkel masks, assuming ideal conditions, we **recommend wearing it for at least 90 s before initiating any fit testing** or it may falsely fail the initial normal breathing test. The amount of time will depend on the user’s breathing rate, tidal volume, number of paticles in the air among other variables.
  o See Section 7 for sample quantitative fit testing results.

### 6.3 Using the full-face snorkel mask as PPE

Please refer to the latest PPE use recommendations from the Centers of Disease Control and Prevention (CDC): https://www.cdc.gov/coronavirus/2019-ncov/hcp/using-ppe.html

#### 6.3.1 Putting on the the full-face snorkel mask

1. Perform hand hygiene using hand sanitizer.
2. Carefully inspect the disinfected snorkel mask, disinfected adapter and pair of filters (for dirt and physical damage to the filter or bayonet fitting). Verify the proper seating of the 3 mushroom valves inside the mask (2 in the mouth compartment and 1 in the exhalation port) and check for any damage. The 2 mouth compartment valves will help minimize CO_2_ rebreathing and facilitate CO_2_ exhaust.
3. Verify the integrity of the putty seal between the adapter and the mask, and that the adapter is pushed in as much as possible to cover the soft plastic seal.
4. Verify the integrity of the inhalation port gaskets.
5. Insert the pair of 3M filters per the manufacturer’s instructions. For most filters it involves aligning the filter bayonet fitting with the adapter’s bayonet fitting, and then rotating ¼ turn clockwise to attach.
6. Put on the mask fully. Adjust and tighten the straps as needed.
7. Perform a suction test: put the mask to your face, press it slightly while inhaling to create suction between the mask and your face. The mask should suction to your face. Try smiling and see if the movement of facial muscle breaks the seal. **IF MASK DOES NOT PASS THE SUCTION TEST, THE MASK SHOULD NOT BE USED.** Some factors that may affect the seal are: facial hair, eyeglasses, long hair, cosmetics, facial scars, face piercings, dentures, jewelry and facial structure. Any hair growth that contacts the sealing surfaces must be shaved clean. Long hair should we secured back to avoid interfering with the seal. Eyeglasses will not permit a good seal and contact lenses should be used. Ocean Reef also provides an optical lens insert for the Aria full face snorkel masks (see **Bill of Materials,** Section 4).
8. Perform hand hygiene using hand sanitizer.

#### 6.3.2 Removing and disinfecting the the full-face snorkel mask

1. Perform hand hygiene using hand sanitizer.
2. Lean head forward into sniff position. Loosen the straps and remove the mask without touching your face.
3. Perform hand hygiene using hand sanitizer.
4. Wear gloves when removing the filters or perform hand hygiene after handling them. Store the filters in a small paper bag with your name to avoid cross-contamination. There are various potential methodologies for disinfecting filters like UV light, but unless carefully evaluated and tested, it is best to avoid these not to compromise the integrity of the filter.
5. The snorkel mask and adapter should be disinfected to avoid contamination of other surfaces. To minimize the risk of damaging or stretching the adapter and snorkel mask seal, unless deemed necessary for a more thorough cleaning, avoid separating the adapter from the mask during daily cleaning.
6. First clean the inner surface with an EPA-approved wipe and dispose of it. Second clean the outer surface with a different wipe and dispose of it. The United States Environmental Protection Agency has a comprehensive list of disinfectants for use against SARS-CoV-2:
  https://www.epa.gov/pesticide-registration/list-n-disinfectants-use-against-sars-cov-2-covid-19 When selecting a disinfectant consider the compatibility with the full-face snorkel mask and adapter materials, contact time, and potential harmful effects of inhaling any fumes.
7. Put the mask into a clean dedicated box or large paper bag with your name.
8. Discard the gloves or perform hand hygiene using a hand sanitizer.
9. At a minimum clean thoroughly the snorkel mask at the end of the work day to avoid contamination of other surfaces and allow for dissipation of any disinfectant fumes in the inner surface of the mask.

#### 6.3.3 When to change the filters

• Too much resistance through the air filters should be avoided as increasing negative pressures inside the mask could increase the likelihood of air leaking in through the exhaust mushroom valve. Some of the 3M filters are rated for industrial applications and in the clinical setting where the air is relatively clean should offer greater longevity especially if the filter supply is limited. Per the manufacturer[13], replace 3M Particulate Filters when:
  o It becomes difficult to breathe comfortably (this will vary from individual to individual).
  o The filter becomes dirty or physical damage occurs.

## 7 Validation and characterization

**Fig. 8:**
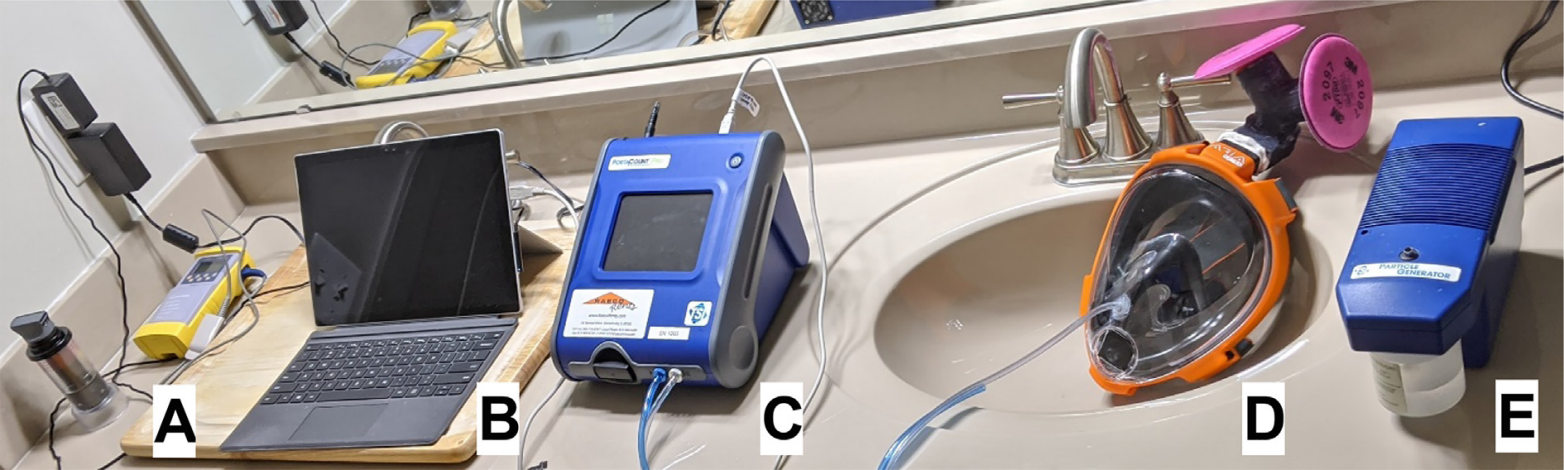
From left to right: **A)** pulse oximeter, **B)** SurfacePro, **C)** PortaCount 8030, **D)** ARIA QR + snorkel mask, and **E)** particle generator 8026. Fit testing setup as described in Section 5.2

The testing equipment consisted of a PortaCount 8030 Respirator Fit Tester (TSI, Minnesota, USA), Particle Generator 8026 Tester (TSI, Minnesota, USA), Surface Pro (Microsoft, Redmond, WA), Nellcor OxiMax N-65 Pulse Oximeter (Medtronic, Minnesota, USA) (Fig. 8), and a gauge manometer (Instrumentation Industries, Inc., Pennsylvania, USA). The PortaCount 8030 was calibrated March 6, 2020 by TSI.

Testing was done in an approximately 9 ft. × 9 ft. room with the particle generator turned on. A 3M 6800 series (3M, St. Paul, MN) full-faced mask served as the benchmark. The three testing sessions were done on three separate days with different ambient particle concentrations. For this reason, fit factors are not directly comparable between sessions. What is important is whether any given fit factor was greater than 500 indicating that it passed the OSHA test.

### 7.1 First testing session

Daily QA for the PortaCount with the particle generator active was performed and passed. In the first testing session we tested the 6600 series 3M mask as our benchmark and three experimental configurations of the Aria QR + mask (medium/large) with a single user using the coated PLA adapter, sealed with putty, with the 3M 2091 filters, and the fit testing method drilling the mask.

These four experimental setups were named as follows:

1. 3M 6800 full face respirator: benchmark
2. Snorkel mask duct tape: In this setup the front plastic cover was removed as shown in this video (skip to 1 min): https://www.youtube.com/watch?v=ewrsJ4lTgj4 (Supplementary Video 1)
  Supplementary video 1, Transforming Snorkeling Mask into non certified PPE - APA - FIRST CONCEPT! UNOFFICIAL!
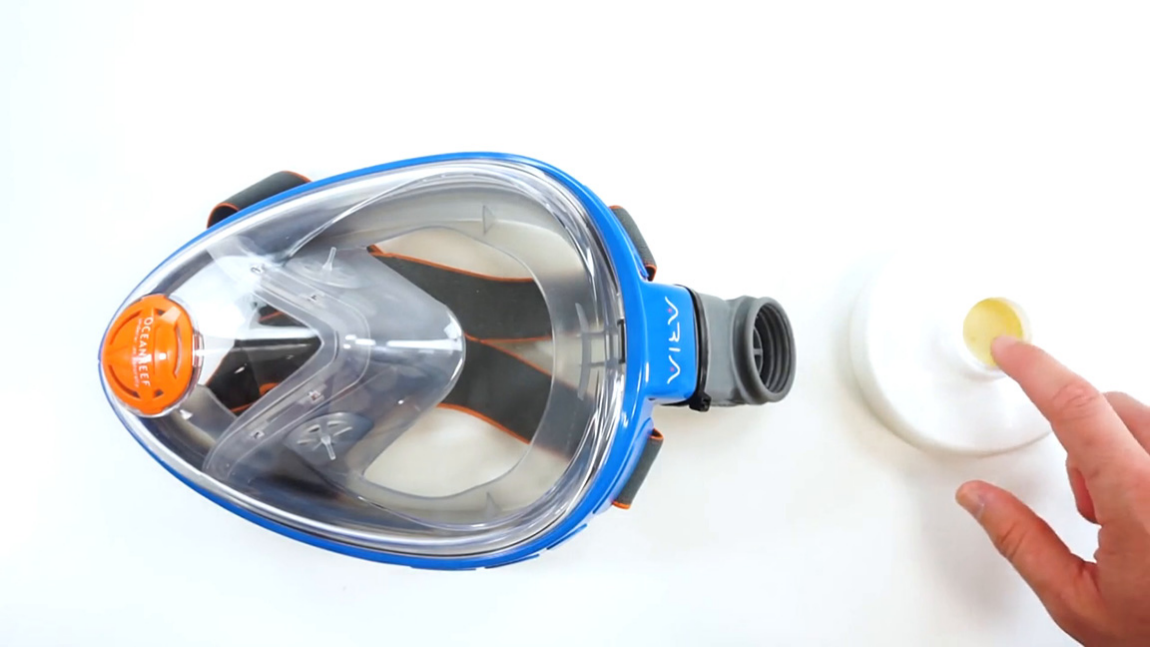
  The mushroom valve by the mouth was also removed. Two square pieces of duct tape covered the resulting opening to seal the port’s opening.
3. Snorkel mask no modifications: This setup preserves the original configuration of the snorkel mask including the mushroom valve and protective cover in front of it.
4. Snorkel mask mouth cover removed: This setup preserves the mushroom valve, but removes the plastic protective cover in front of it as shown in the previous video.

#### 7.1.1 3M 6800 full face respirator results

The 6800 series 3M full face respirator served as our benchmark and reached its maximum fit factor in 1:15 min. O_2_ Saturation remained stable. Real time test results can be seen here:

https://drive.google.com/file/d/1hY2ZT3B8HeySWEC7M7wPVEo2ACfaUtaZ/view?usp=sharing

(Supplementary Video 4)

Supplementary video 4, 7.1.1 3M 6800 full face respirator real time results
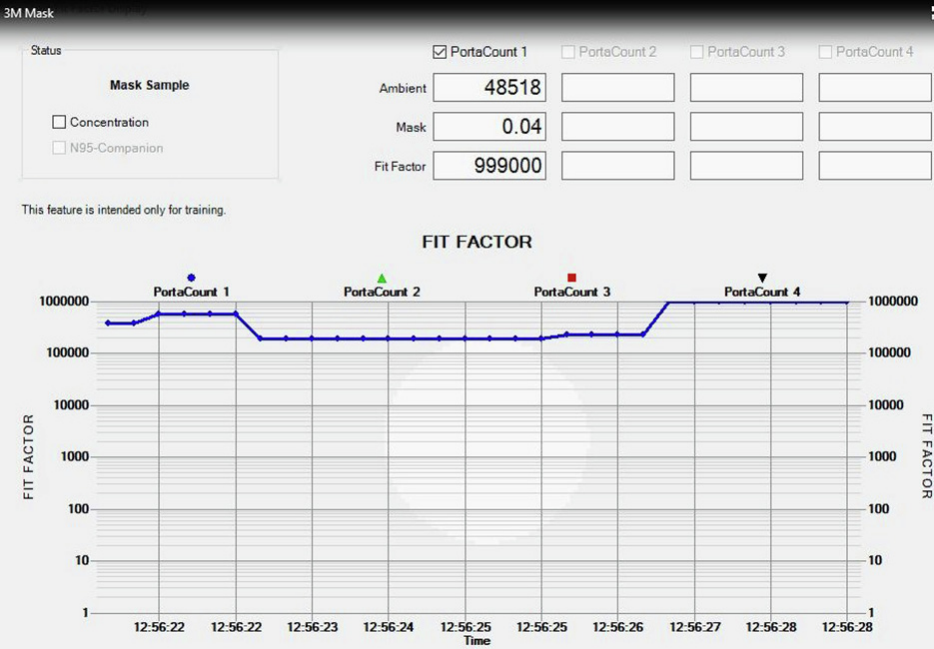

The 3M mask passed the fit test results with a fit factor of 333,867. Fogging or humidity were not an issue.

#### 7.1.2 Snorkel mask duct tape results

This configuration reached its maximum fit factor in approximately 2:11 min. O_2_ Saturation remained stable. Real time test results can be seen here:

https://drive.google.com/file/d/1ETWlNk0VjiZnSMaRXsm5R3HmVk0Zrd-M/view?usp=sharing

(Supplementary Video 5)

Supplementary video 5, 7.1.2 Snorkel mask duct tape real time results
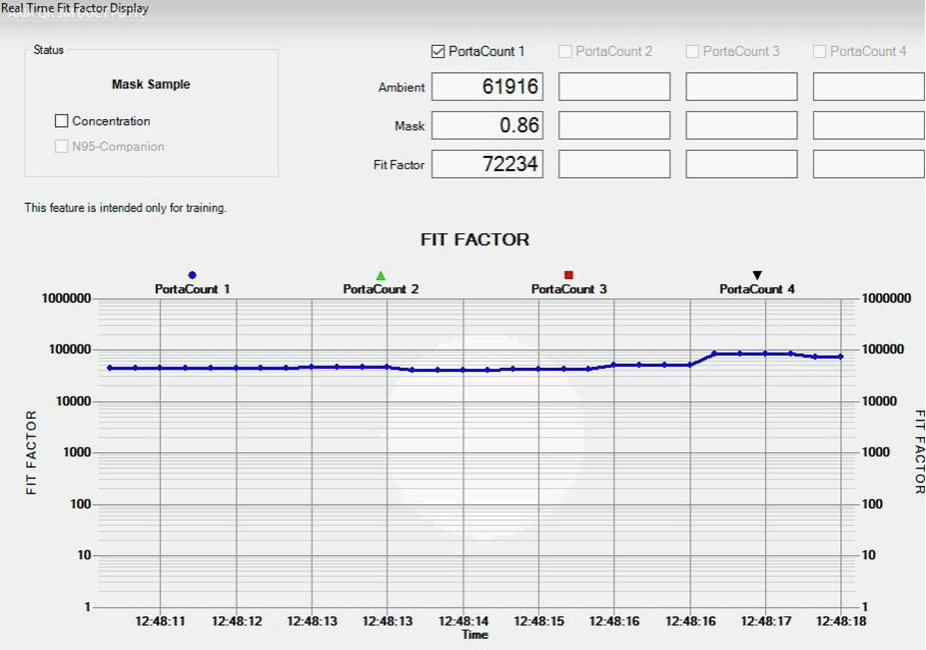

This configuration passed the fit test results with a fit factor of 32,281 (Table 1). Increased humidity decreased the comfort of the mask although fogging was minor.

**Table 1:** First testing session. Fit testing results for the Aria QR + mask (medium/large) using the coated PLA adapter, sealed with putty, with the 3M 2091 filters, and the fit testing method drilling the mask (Fig. 5).

Mask configuration	3M 6800 full face respirator	Snorkel mask duct tape	Snorkel mask no modifications	Snorkel mask mouth cover removed
Normal breathing	297,913	44,910	18,511	1123
Deep breathing	288,127	40,394	27,612	1412
Head side to side	442,017	63,628	38,360	1541
Head up and down	349,546	66,749	63,259	1234
Talking	266,729	21,200	7933	1953
Bending over	326,150	12,676	7534	929
Normal breathing	454,406	76,511	16,065	593
Overall fit factor	333,867	32,281	15,448	1105
Fit Test*	Pass	Pass	Pass	Pass

* OSHA fit factor passing value is 500 or greater.

#### 7.1.3 Snorkel mask no modifications results

This configuration reached its maximum fit factor in approximately 1:56 min. O_2_ Saturation remained stable. Real time test results can be seen here:

https://drive.google.com/file/d/1Hpwc90kCozlu2EnfLrXCWGt5bk2bqQw8/view?usp=sharing

(Supplementary Video 6)

Supplementary video 6, 7.1.3 Snorkel mask no modifications real time results
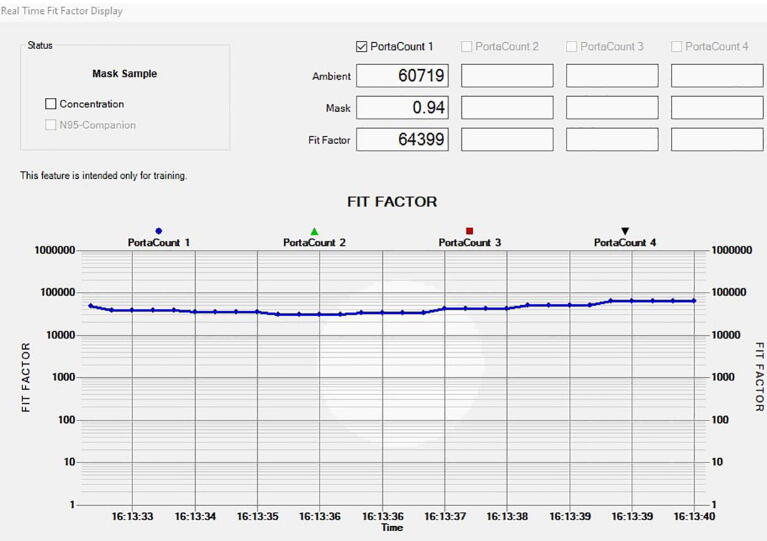

This configuration passed the fit test results with a fit factor of 15,448 (Table 1). Fogging or humidity were not an issue. Further evaluation of this configuration generated during inspiration a negative pressure of 2 cm of water and no positive pressure during exhalation.

*Subjective user experience*: A radiation therapist wore the non-modified snorkel mask from 9 AM to 3 PM while performing daily work activities which require an increased level of exertion while positioning and moving patients to the treatment couch of the linear accelerator. She took off the mask for lunch and for a break to have a drink. She treated between 25 and 30 patients that day. Visibility was great and comfort better than other PPE she had used. Near the end of the daily with increased physical activity she felt some breathing discomfort. She measured the O_2_ sat at the time which was 100%. Overall her feedback was that it was a comfortable option she could tolerate for prolonged periods of time. The only negative feedback was that the patients and the other therapists had a hard time hearing her, requiring speaking up or using hand gestures.

#### 7.1.4 Snorkel mask mouth cover removed results

This configuration reached its maximum fit factor in approximately 1:08 min. O_2_ Saturation remained stable. Real time test results can be seen here:

https://drive.google.com/file/d/1kPSIH0cFRrhGwltuDOD-RODMJAn9U_OG/view?usp=sharing

(Supplementary Video 7)

Supplementary video 7, 7.1.4 Snorkel mask mouth cover removed real time results
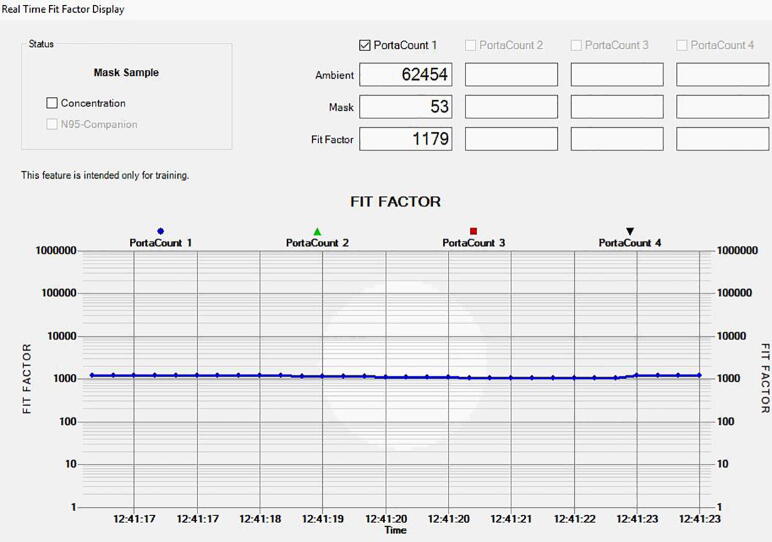

This configuration passed the fit test results with a fit factor of 1105 (Table 1). Fogging or humidity were not an issue.

### 7.2 Second testing session

A second testing session on a different day tested additional 3M filters, the preferred of the previous configurations (snorkel mask with no modifications), the Aria Classic mask (medium/large), the higher quality Somos GP resin (DSM) adapter that did not require sealing, the fit testing method with the modified adapter, and the snorkel mask with no modifications configuration with a single user. Daily QA for the PortaCount with the particle generator on was performed and passed (Table 2.).

**Table 2:** Second testing session. Fit testing results for the Aria Classic mask (medium/large) using the Somos GP resin (DSM) adapter, sealed with putty, the fit testing method with the modified adapter, and the snorkel mask with no modifications configuration (see Fig. 6). Different testing session compared to Table 1.

3 M filter	2091	2071	7093CN	5N11**
Normal breathing	5340	8870	5611	130
Deep breathing	5481	7312	6066	104
Head side to side	4908	8880	5928	140
Head up and down	4470	8946	5835	149
Talking	3092	5783	5945	105
Bending over	2302	4231	3602	95
Normal breathing	3880	8527	5560	126
Overall fit factor	3862	6997	5348	118
Fit Test*	Pass	Pass	Pass	Fail

* OSHA fit factor passing value is 500 or greater.

** 3M 603 filter adapter and 501 filter retainer.

### 7.3 Third testing session

A third testing session on a different day fit tested various radiotherapy clinic staff (three radiation therapists, a nurse, and a physician) with different combinations of masks and filters. Daily QA for the PortaCount with the particle generator active was performed and passed (Table 3.).

**Table 3:** Third testing session. Radiotherapy clinic staff (3 radiation therapists, a nurse, and a physician) testing results using the Somos GP resin (DSM) adapter, sealed with putty, the fit testing method with the modified adapter, and the snorkel mask with no modifications configuration.

Mask	Aria UNO	Aria Classic	Aria Classic	Aria QR+	Aria QR+
Size	S/M	S/M	L/XL	M/L	M/L
3M filter	2071	2091	SP71**	2091	2097
Normal breathing	12,125	11,767	2350	29,481	14,182
Deep breathing	7951	11,431	2598	18,873	27,623
Head side to side	8183	893	2070	28,781	56,895
Head up and down	15,326	2521	1967	461	41,572
Talking	1552	1517	951	2860	2339
Bending over	3891	2713	1445	97	60,473
Normal breathing	8505	6693	2050	31,736	108,685
Overall fit factor	4949	2442	1736	543	11,637
Fit Test*	Pass	Pass	Pass	Pass	Pass
Notes	passed on 2nd try after pulling back top straps			cosmetics	

* OSHA fit factor passing value is 500 or greater.

** using 3M 603 filter adapter and 501 filter retainer, due to the clamshell design more prone to user error.

## 8 Discussion

Although subjective qualitative fit testing can reveal promising PPE air filtration solutions, quantitative testing will ultimately reveal the effectiveness of any given solution. Our results are comparable to results obtained from commercial systems [14] and offer an alternative when a commercial full face respirator is unavailable especially during a pandemic PPE shortage. An advantage of the full-faced snorkel mask design is that it serves two critical purposes: eye and face protection, and high quality air filtration to protect against SARS-CoV-2. Users should pay attention to facial hair, eyeglasses, and other factors that could affect the mask’s seal. Even the smallest of air leaks could significantly compromise the fit factor.

Critical to the success of the project were using the commercial filters and inhalation port gaskets, a completely airtight adapter, leaving the plastic part covering the mouth of the mask, and sealing the connection between the mask and adapter. Any design should minimize dead space in the adapter and/or mask, especially in hermetically sealed systems, which can result in CO_2_ rebreathing and adverse symptoms.

Regarding the two fit testing methodologies, drilling the hole on the mask methodology is very convenient for prototyping adapters. Once a prototype design is finalized and ready to implement, creating an adapter exclusively for quantitative fit testing is desirable as the specific user’s mask won’t need to be damaged.

Although the PortaCount’s real time testing is not part of the overall fit factor testing. We did find it useful during prototyping to quickly determine if a design had potential for passing the fit testing versus doing the lengthier 8 exercise overall fit testing. The real time testing also provided us a valuable insight: since the volume that initially has to be filtered is larger for the snorkel mask (mouth compartment + face compartment) versus the commercial mask (mouth compartment only), we had to wait longer before initiating the full-faced snorkel mask testing.

The second testing session showed that the 3M 2091, 2071, and 7093CN filters with different filtration capabilities and two different filter designs passed. Different from the first testing session, we used the Aria Classic mask using the Somos GP resin (DSM) adapter, the fit testing method with the modified adapter, and the snorkel mask with no modifications configuration. We showed the feasibility of a fit testing method that does not damage the mask. The 5N11 filter encased in clamshell comprised of the 603 filter adapter and 501 filter retainer failed, although it would have passed the OSHA fit factor passing value is 100 or greater for half-face masks.

The third testing session involved 5 different healthcare staff using a combination of snorkel mask models by the same manufacturer and different filters. The head strap design of the Aria UNO requires pulling back the top straps for a better seal, and is more prone to user error. The design of the straps tends to generate less pressure on the top of the mask which can result in poor fit factors. One user’s use of facial cosmetics may have resulted in < 500 fit factors when tilting the head up and down, and bending over. Although the design of the 5N11 N95 and 5P71 P95 filters is similar (Fig. 2), only the 5P71 P95 filter was able to achieve a fit factor greater than 500 despite additional attempts testing the 5N11 not shown. Both filters were encased in the 603 filter adapter and 501 filter retainer clamshell design which is less desirable as it requires additional parts and is more prone to user error. Since the 5P71 meets NIOSH **P95**, while the 5N11 meets NIOSH **N95** criteria, perhaps this could explain the difference in fit factors. Another staff with a beard was tested but the results were not included because, as expected, his beard impaired achieving a fit factor greater than 500.

The snorkel mask manufacturer has various models that share the same basic design, but with different price points. Nevertheless, we prefer the Aria Classic and QR + as the strap design is less prone to user error. Our solution is a cost-effective and environmentally effective solution for creating a full-face PPE mask replacement. The masks are effectively a reusable face shield, and the industrial 3M filters should have a long useful life in the relatively clean air in most hospitals. The generated waste is reduced and the effectiveness of this solution exceeded OSHA standards. If available, a professional 3M mask should be preferred as it is specifically engineered to use the 3M filters, directly filters the air entering the mouth/nose compartment (instead of the face compartment first and then the mouth/nose compartment), and has been thoroughly tested for industrial applications.

At the beginning of the pandemic, the main barrier to implement this solution was obtaining the filters due to the high demand, but their availability has been increasing. Price gouging and counterfeit filters were additional problems in the beginning. The Aria snorkel masks are readily available, as well as the other materials required to implement this project.

Best practices regarding the disinfection process of the mask and adapter need to be further explored, and we provide a starting point for exploring many of the available potential options. For disinfection, we explored UVC light with 254 nm wavelength and used an ILT770-NB (International Light Technologies, Inc., Peabody, MA) narrow band 254 nm light meter for measurements. A UV sterilization system needs to deliver at least 1 J/cm^2^ is reportedly sufficient to eliminate SARS-CoV-2 [15]. We found that reliable UVC disinfection was not practical because not only the opaque plastic, but the clear visor, were very efficient at blocking the UVC resulting in shadows in the mask’s internal surfaces. Due to the inherent uncertainties brought by shadowing that could protect contaminated areas (or at the very least fincrease the time to decontaminate the mask), other methods of disinfection seem more practical and reliable.

Creating a universal adapter that could easily secure a validated filter material that could achieve at least N95 filtration would make this more accessible to the general public. However, this is challenging for the following reasons:

1. Creating an airtight filter seal with an easy to use mechanism is challenging.
2. Creating a filter with N95 properties with more accessible materials has proven difficult once rigorous aerosol testing is performed.
3. Breathability and filter effectiveness are also a function of the filter’s surface area. An N95 mask has an average surface area from 110 to 135 cm^2^. In contrast, the surface area of two 3M P100 filters is 380 cm^2^. Larger surface areas help slow down particles so that they can be more easily trapped, and improve breathability.

We also created an adapter for Honeywell P100 filters, but given that these are threaded filters, getting an airtight seal was very difficult in our consumer grade 3D printer. We had more success with a professional print in Somos GP and using Teflon tape, but the connection did not seem robust or reproducible enough for daily use.

Finally, we want to emphasize that potential users should do quantitative testing before assuming their prints will achieve similar results. Users should understand that this is an off-label application that is not FDA cleared and should be used at your own risk.

## 9 Conclusion

The modified full-faced snorkel mask tested solved two critical PPE problems in the current COVID-19 crisis: eye and face protection, and high quality air filtration to protect against SARS-CoV-2. The solution exceeded the OSHA requirements for a full faced mask in quantitative testing, and should be further evaluated as a PPE alternative in the current COVID-19 related PPE shortage.

## Declaration of Competing Interest

The authors (Kyle Nicholson, Ashley Henke-Adams, Daniel M. Henke, Alexxai V. Kravitz, and Hiram A. Gay) declare that they have no known competing financial interests or personal relationships that could have appeared to influence the work reported in this paper.

## Appendix A Supplementary data

Supplementary, OceanReef filter adapter - 3M
